# Supplementary material for: Antibacterial Efficacy of N-(4-methylpyridin-2-yl) Thiophene-2-Carboxamide Analogues against Extended-Spectrum-β-Lactamase Producing Clinical Strain of Escherichia coli ST 131
Source: Molecules. 2023 Mar 31;28(7):3118. doi: 10.3390/molecules28073118 (PMC10095758; doi:10.3390/molecules28073118)
Supplement: Supplementary file 1 [file molecules-28-03118-s001.zip › molecules-2134173-supplementary.pdf]

Supplementary Data

Anti-bacterial efficacy of *N*-(4-methylpyridin-2-yl) thiophene-2-carboxamide analogues against extended-spectrum-β-lactamase producing clinical strain of *Escherichia coli* ST 131

Gulraiz Ahmad [1], Aqsa Khalid [2], Muhammad Usman Qamar [3] , Nasir Rasool [1,*], Malik Saadullah [4], Muhammad Bilal[5], Majed A. Bajaber [6], Ahmad J. Obaidullah [7], Hadil Faris Alotaibi [8], Jawaher M. Alotaibi [7]

[1] Department of Chemistry, Government College, University Faisalabad, 38000, Pakistan; gulchemist35@gmail.com

[2] School of Interdisciplinary Engineering & Science (SINES), National University of Sciences and Technology (NUST), Islamabad 44000, Pakistan; aqsa.khalid30@yahoo.com

[3] Department of Microbiology, Faculty of Life Sciences, Government College University Faislabad; musmanqamar@gcuf.edu.pk

[4] Department of Pharmaceutical Chemistry Government College University Faisalabad; maliksaadullah@gcuf.edu.pk

[5] School of Chemistry and Chemical Engineering, Shandong University, Jinan 250100, China; muhammadbilalgcuf@gmail.com

[6] Chemistry Department, Faculty of Science, King Khalid University, P.O. Box 9004, Abha 61413, Saudi Arabia. mb@kku.edu.sa

[7] Department of Pharmaceutical Chemistry, College of Pharmacy, King Saud University, P.O. Box 2457, Riyadh 11451, Saudi Arabia; aobaidullah@ksu.edu.sa (A.J.O.), 441203066@student.ksu.edu.sa (J.M.A.)

[8] Department of Pharmaceutical Sciences, College of Pharmacy, Princess Nourah bint AbdulRahman University, Riyadh 11671, Saudi Arabia; hfalotaibi@pnu.edu.sa

* Correspondence: nasirrasool@gcuf.edu.pk; Tel.: +92-332-7491790 (N.R.)

Table of Contents

Table S1: Energy score and Root Mean Square Deviations (RMSDs) of selected docked poses ……………………..…………………………………………………………………**S3**

Figure S1: Protein ligand interaction analysis of ligands with Beta-lactamase from Escherichia coli (6N9K) protein (A) 6N9K interactions with **4b** compound (B) 6N9K interactions with **4d** compound (C) 6N9K interactions with 4e compound (D) 6N9K interactions with **4f** compound (E) 6N9K interactions with **4g** compound (F) 6N9K interactions with **4h** compound ……………………………………………..……………… **S4**

Figure S2: Docking interaction of tazobactam reported as β-lactamase inhibitors with CTX-M-15 enzyme (PDB ID: 7BDS) ………………………………………………….………… **S5**

Figure S3: Protein ligand interaction analysis of ligands with Beta-lactamase from Klebsiella pneumonia having expression in Escherichia coli (7BDS) protein (A)7BDS interactions with **4b** compound (B) 7BDS interactions with **4d** compound (C) 7BDS interactions with **4e** compound (D) 6N9K interactions with **4f** compound (E7BDS interactions with **4g** compound (F) 7BDS interactions with **4h** compound ………………………………………………… **S6**

Figure S4: ^{1}H NMR spectrum of compound **3** **S7**

Figure S5: ^{13}C NMR spectrum of compound **4a** **S7**

Figure S6: ^{1}H NMR spectrum of compound **4b** **S8**

Figure S7: ^{1}H NMR spectrum of compound **4c** **S8**

Figure S8: ^{1}H NMR spectrum of compound **4d** **S9**

Figure S9: ^{1}H NMR spectrum of compound **4e** …………... **S9**

Figure S10: ^{1}H NMR spectrum of compound **4f** **S10**

Figure S11: ^{13}C NMR spectrum of compound **4f** …………... **S10**

Figure S12: ^{1}H NMR spectrum of compound **4g** **S11**

Figure S13: ^{13}C NMR spectrum of compound **4g** **S11**

Figure S14: ^{1}H NMR spectrum of compound **4h** **S12**

Table S1: Energy score and Root Mean Square Deviations (RMSDs) of selected docked poses

Ligands	6N9K		
	**S-score**	**RMSD**	**Interacting Residues**
**4a**	-5.2	3.3	Hydrogen bonding: R257<br>Hydrophobic: N255, D258
**4b**	-4.6	1.5	Hydrogen bonding: Y219, K358<br>Hydrophobic: D63, A65, A224, W217
**4c**	-5.3	1.6	Hydrogen bonding: Y219, K358<br>Hydrophobic: T219, W217, A224
**4d**	-4.7	2.08	Hydrogen bonding: K358<br>Hydrophobic: P42, I64
**4e**	-4.8	3.0	Hydrogen bonding: G222<br>Hydrophobic: P42, Y219, I64, A224
**4f**	-4.6	2.8	Hydrophobic: P42, I64, Y219, A224
**4g**	-4.2	1.9	Hydrophobic: P42, I64, Y219, A224
**4h**	-5.0	2.0	Hydrophobic: I64, A65, W217, Y219, A224
**7BDS**			
**Tazobactum**	-5.4	2.78	Hydrogen bonding: R44, E64, R65, A172, T264
**4a**	-6.6	1.5	Hydrogen bonding: S130, S220, G236<br>Hydrophobic: Y105, A219, R274
**4b**	-6.4	2.7	Hydrogen bonding: S130, S220, G236<br>Hydrophobic: Y105, A219, S237, R274
**4c**	-6.5	1.3	Hydrogen bonding: S130, P167<br>Hydrophobic: S237
**4d**	-6.5	1.6	Hydrogen bonding: S130<br>Hydrophobic: Y105, A219, S237
**4e**	-6.4	3.6	Hydrogen bonding: N132<br>Hydrophobic: Y105, N170, S237, G238,
**4f**	-6.4	1.9	Hydrogen bonding: P167, N170, T216<br>Hydrophobic: S237, G238
**4g**	-6.3	2.3	Hydrogen bonding: K73, N132<br>Hydrophobic: Y105, A219, S237, R274
**4h**	-6.4	3.0	Hydrogen bonding: S130, G236, S220<br>Hydrophobic: Y105, P167, A219, S237, R274

*S-score represents the binding energy of the protein-ligand complex

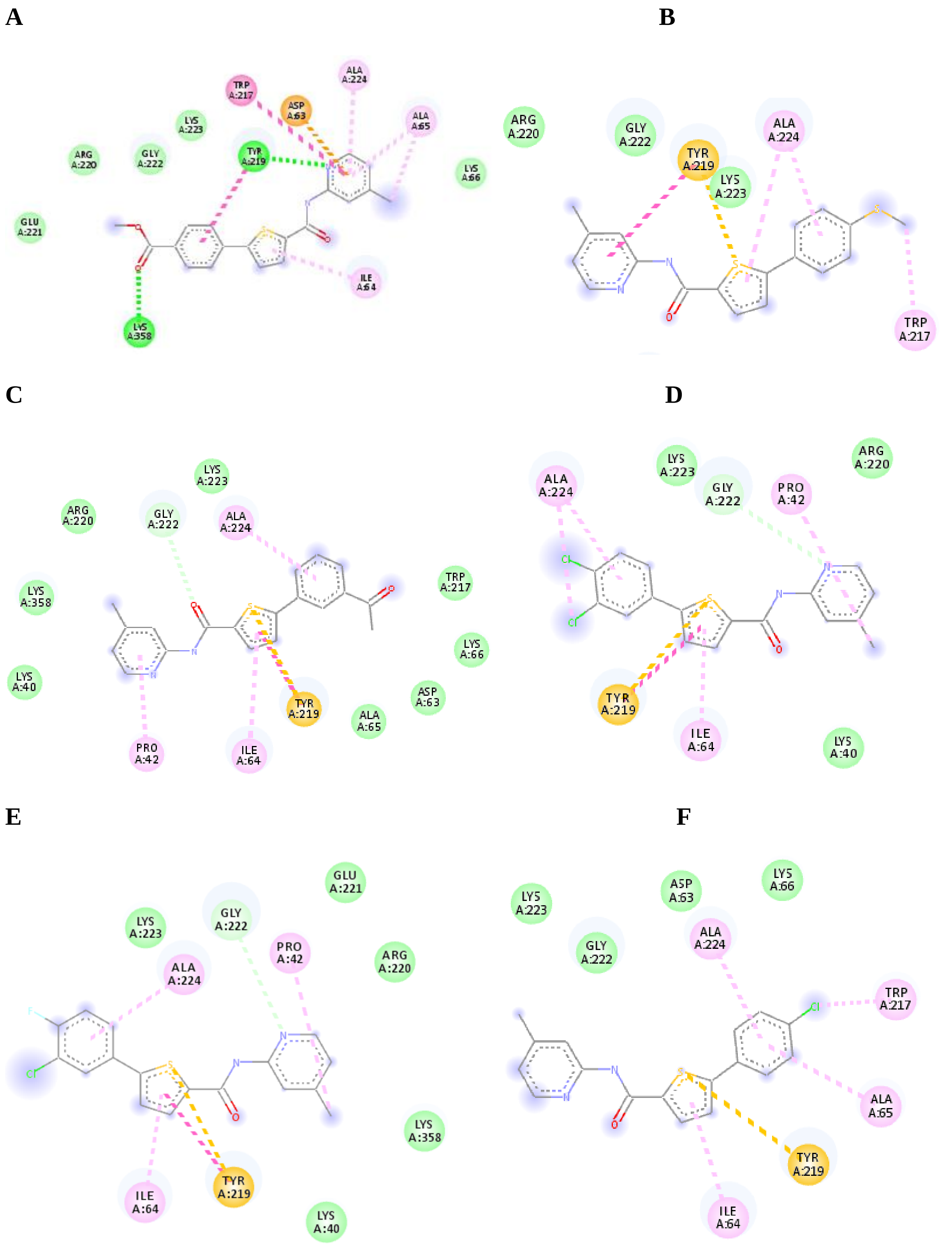

Figure S1: Protein ligand interaction analysis of ligands with Beta-lactamase from Escherichia coli (6N9K) protein (A) 6N9K interactions with **4b** compound (B) 6N9K interactions with **4d** compound (C) 6N9K interactions with **4e** compound (D) 6N9K interactions with **4f** compound (E) 6N9K interactions with **4g** compound (F) 6N9K interactions with **4h** compound

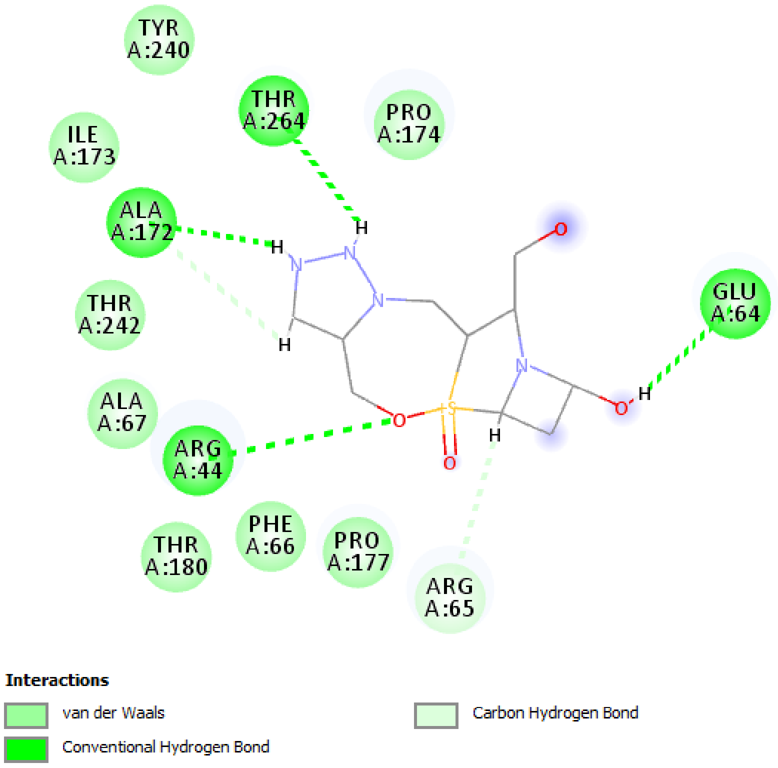

Figure S2: Docking interaction of tazobactam reported as β-lactamase inhibitors with CTX-M-15 enzyme (PDB ID: 7BDS).

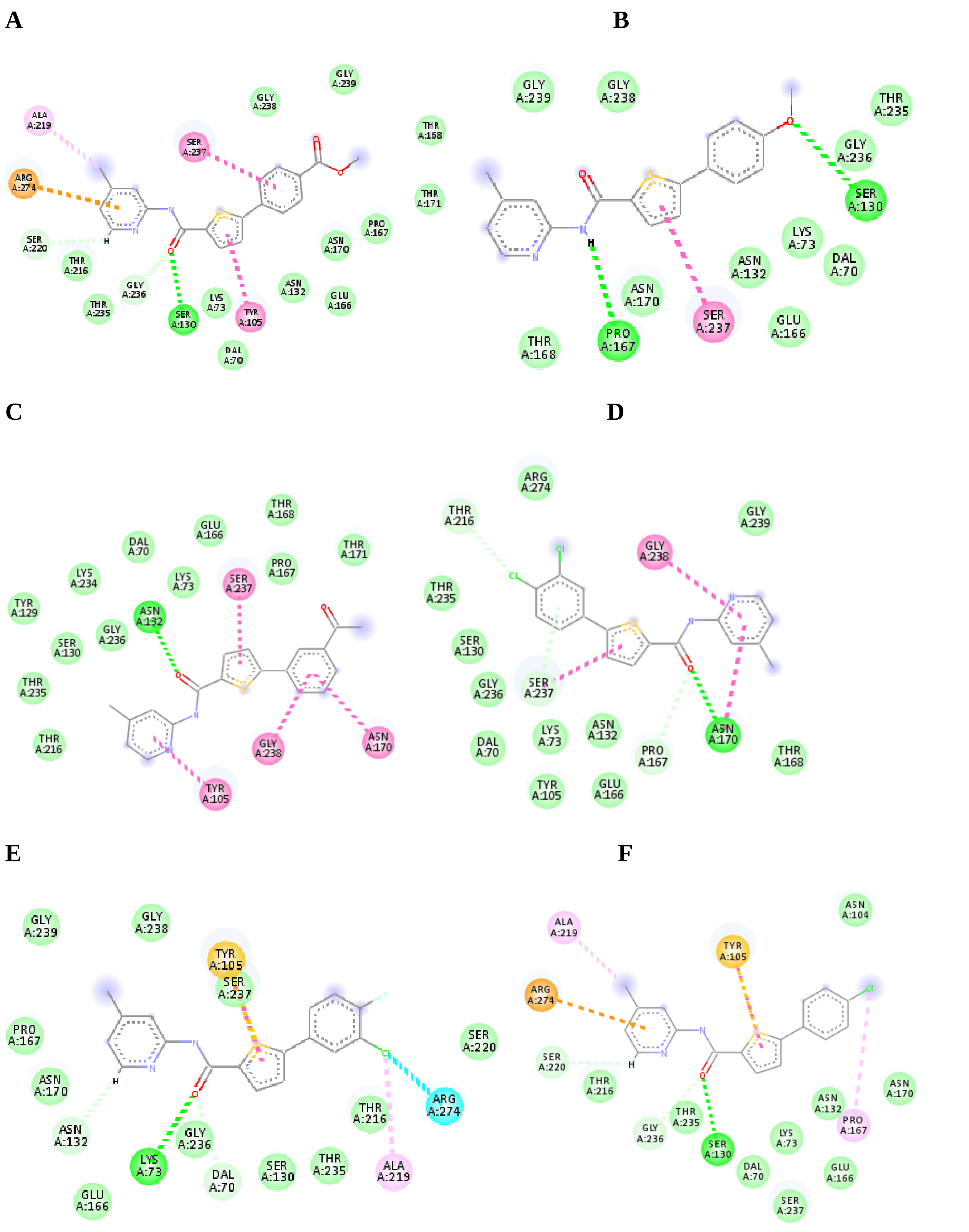

Figure S3: Protein ligand interaction analysis of ligands with Beta-lactamase from Klebsiella pneumonia having expression in Escherichia coli (7BDS) protein (A)7BDS interactions with **4b** compound (B) 7BDS interactions with **4d** compound (C) 7BDS interactions with **4e** compound (D) 6N9K interactions with **4f** compound (E7BDS interactions with **4g** compound (F) 7BDS interactions with **4h** compound

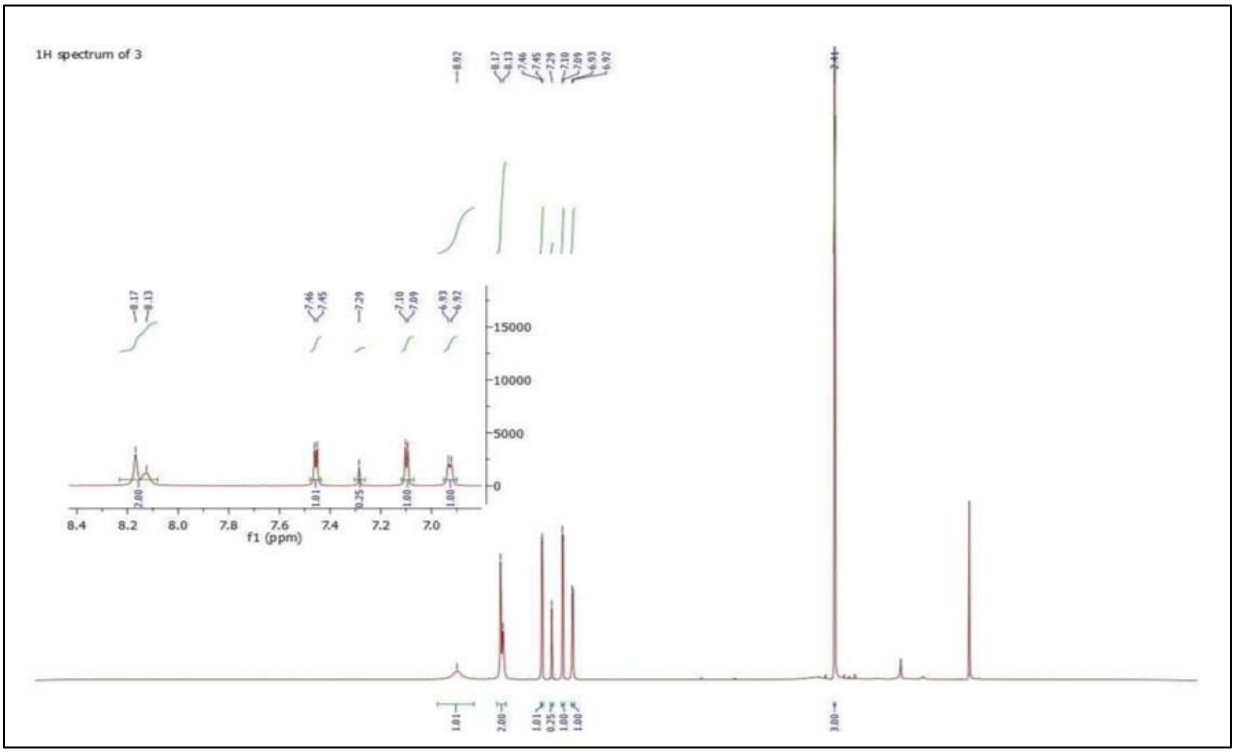

Figure S4: ^{1}H NMR (400 MHz, $CDCl_3$) of compound **3**.

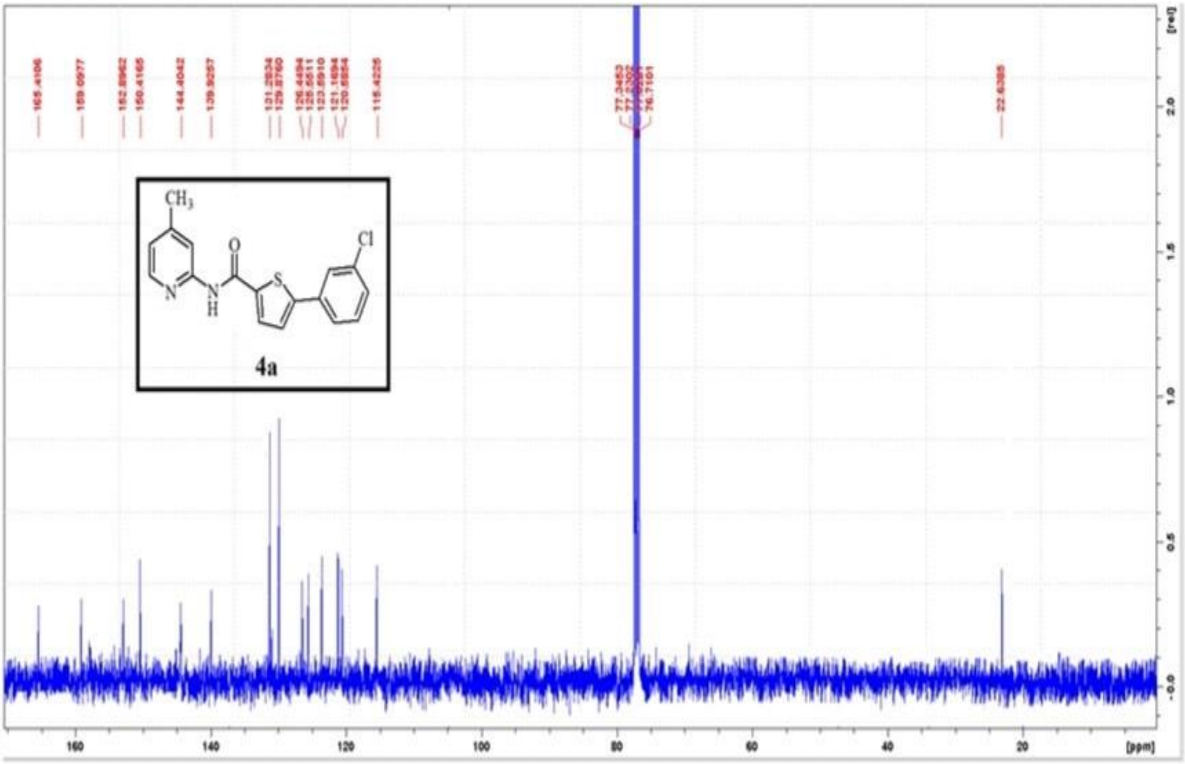

Figure S5: ^{13}C NMR (100 MHz, $CDCl_3$) of compound **4a**.

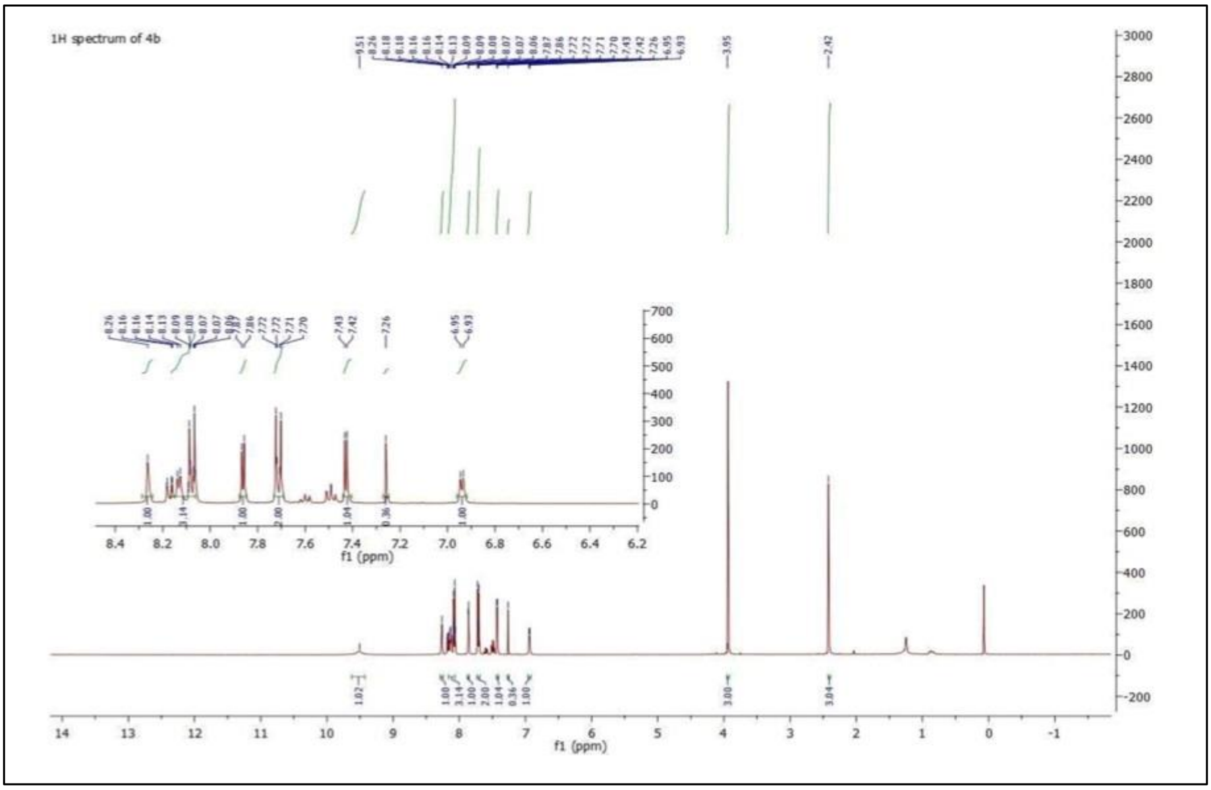

Figure S6: ^{1}H NMR (400 MHz, $CDCl_3$) of compound **4b**.

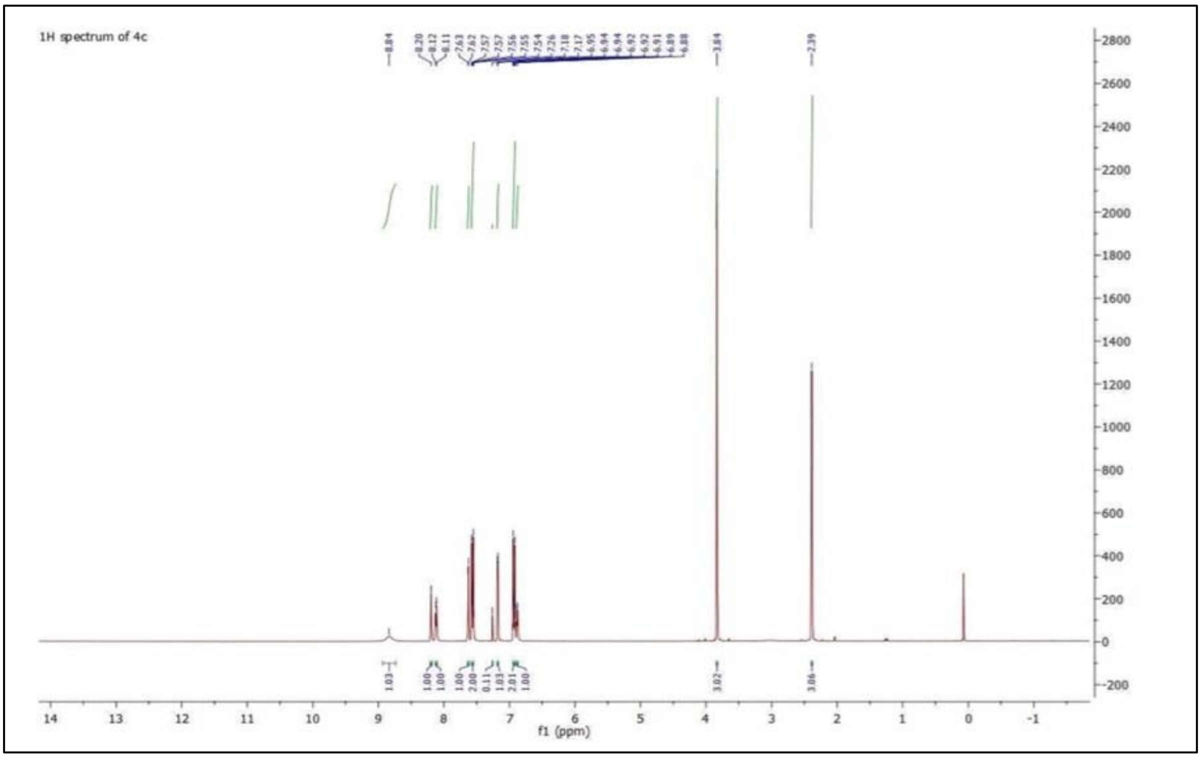

Figure S7: ^{1}H NMR (400 MHz, $CDCl_3$) of compound **4c**.

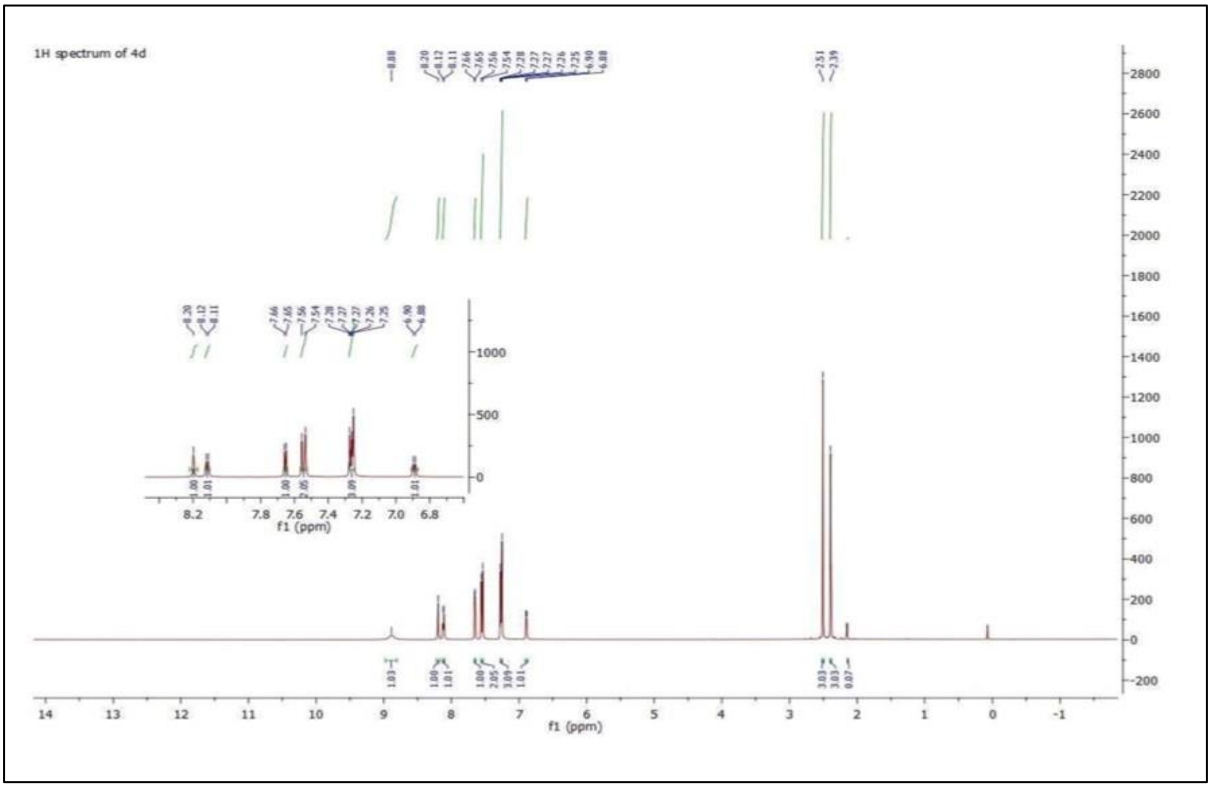

Figure S8: ^{1}H NMR (400 MHz, $CDCl_3$) of compound **4d**.

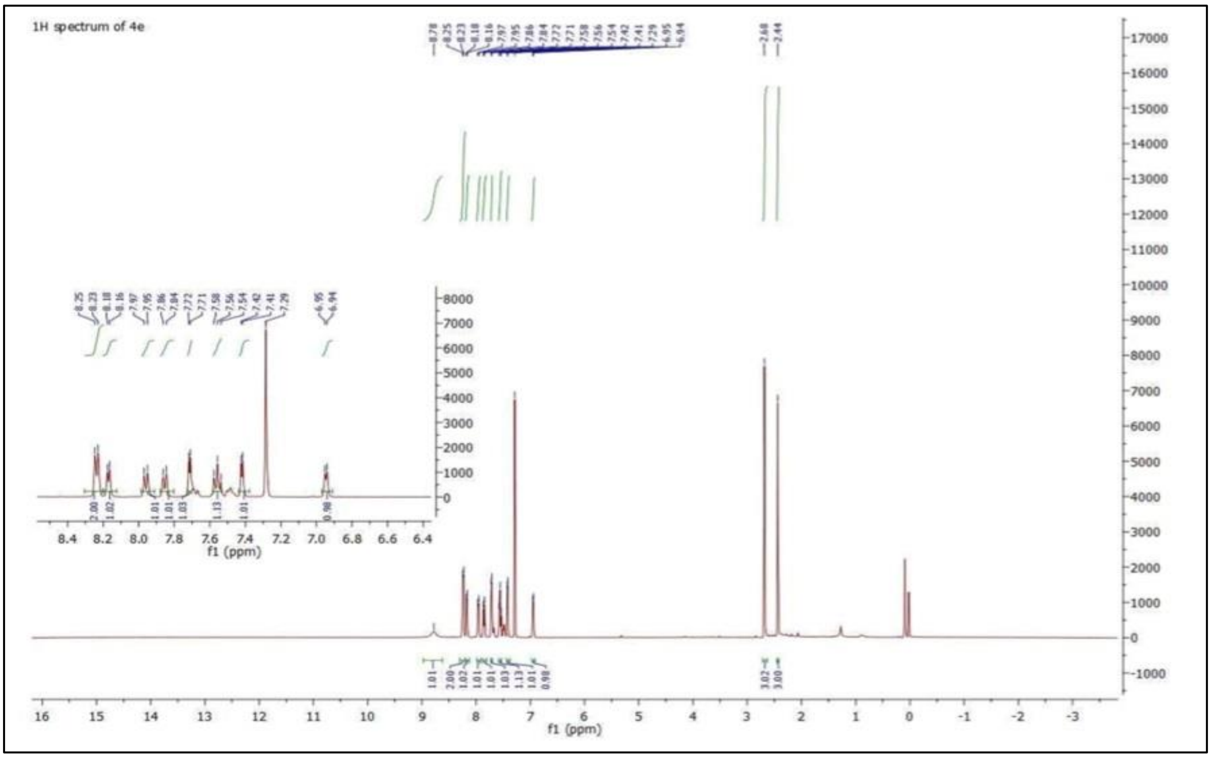

Figure S9: ^{1}H NMR (400 MHz, $CDCl_3$) of compound **4e**.

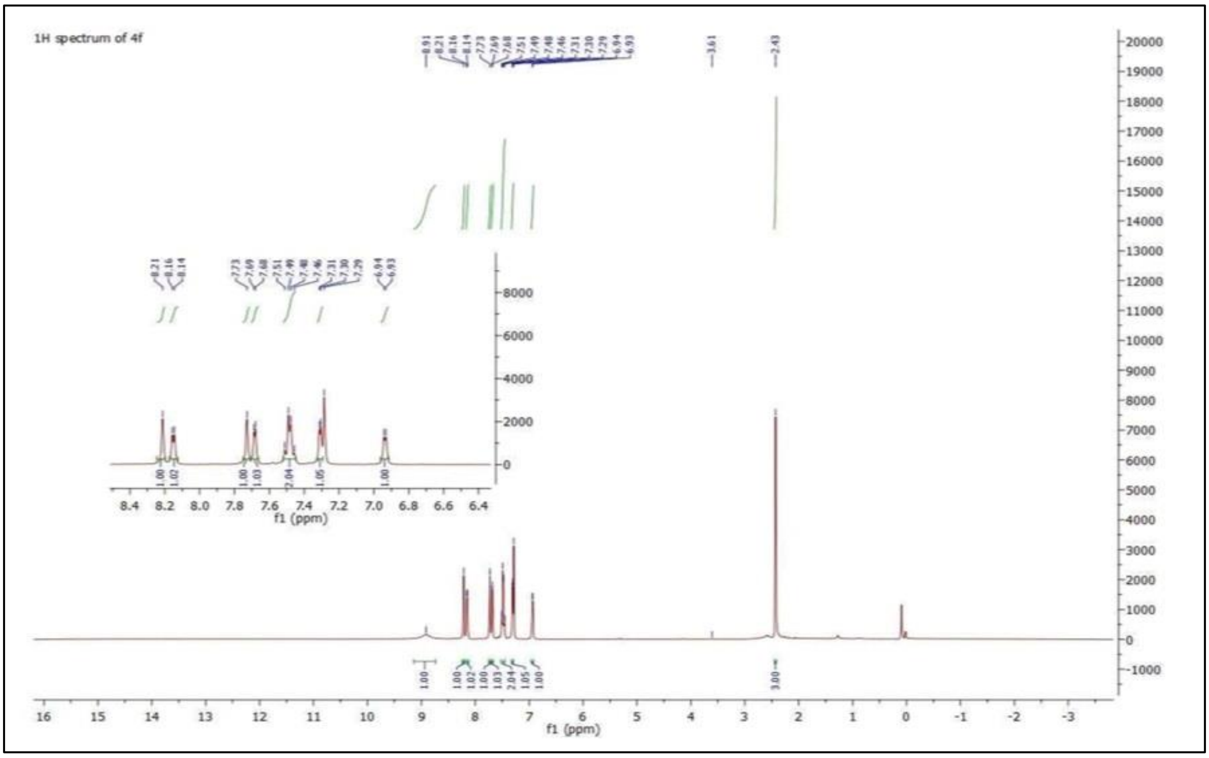

Figure S10: ^{1}H NMR (400 MHz, $CDCl_3$) of compound **4f**.

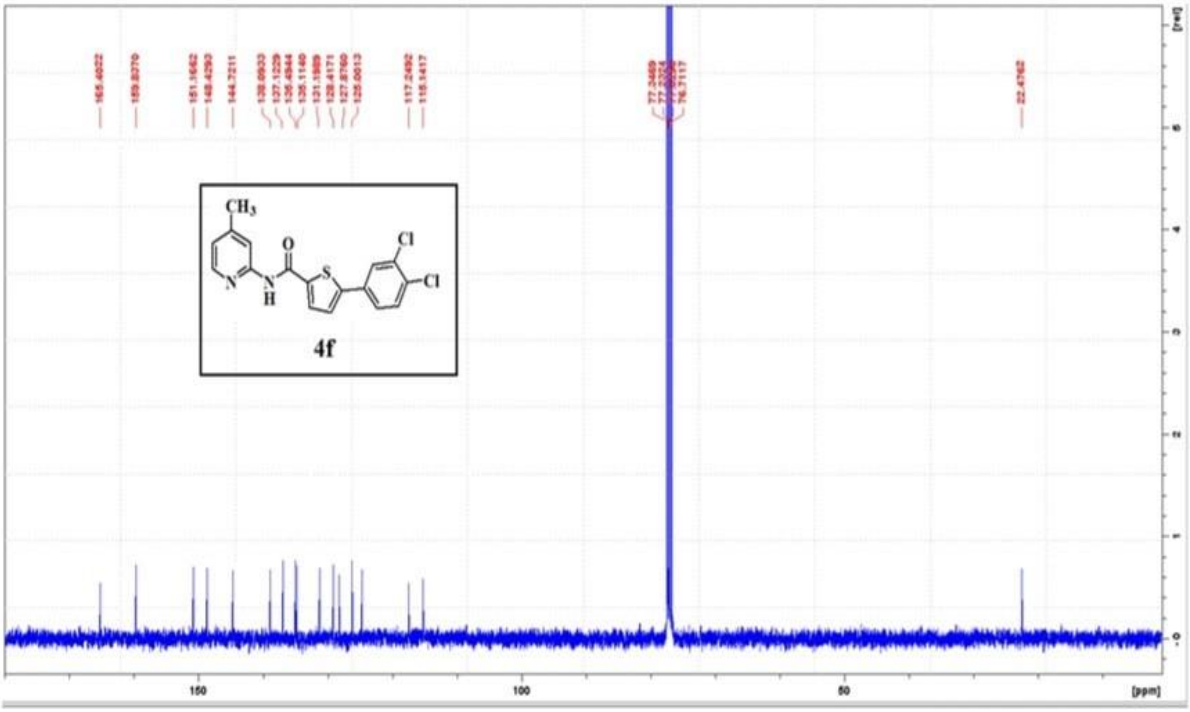

Figure S11: ^{13}C NMR (100 MHz, $CDCl_3$) of compound **4f**.

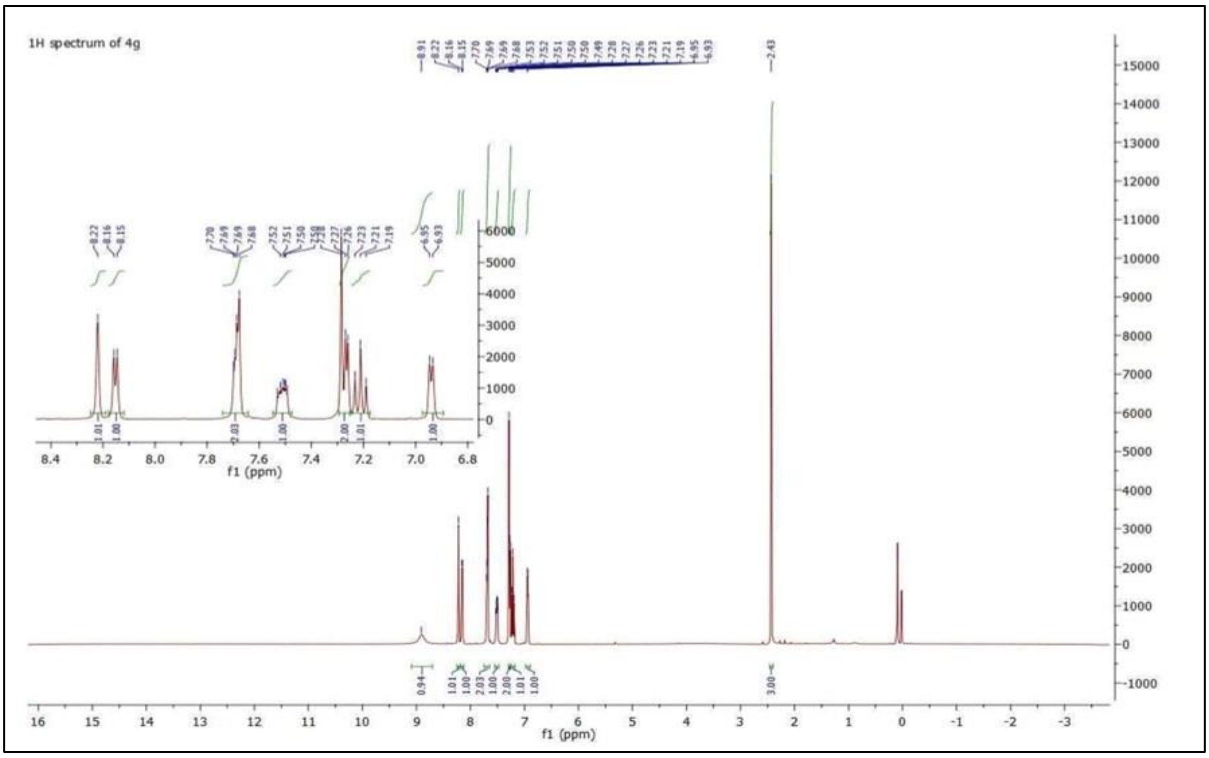

Figure S12: ^{1}H NMR (400 MHz, $CDCl_3$) of compound **4g**.

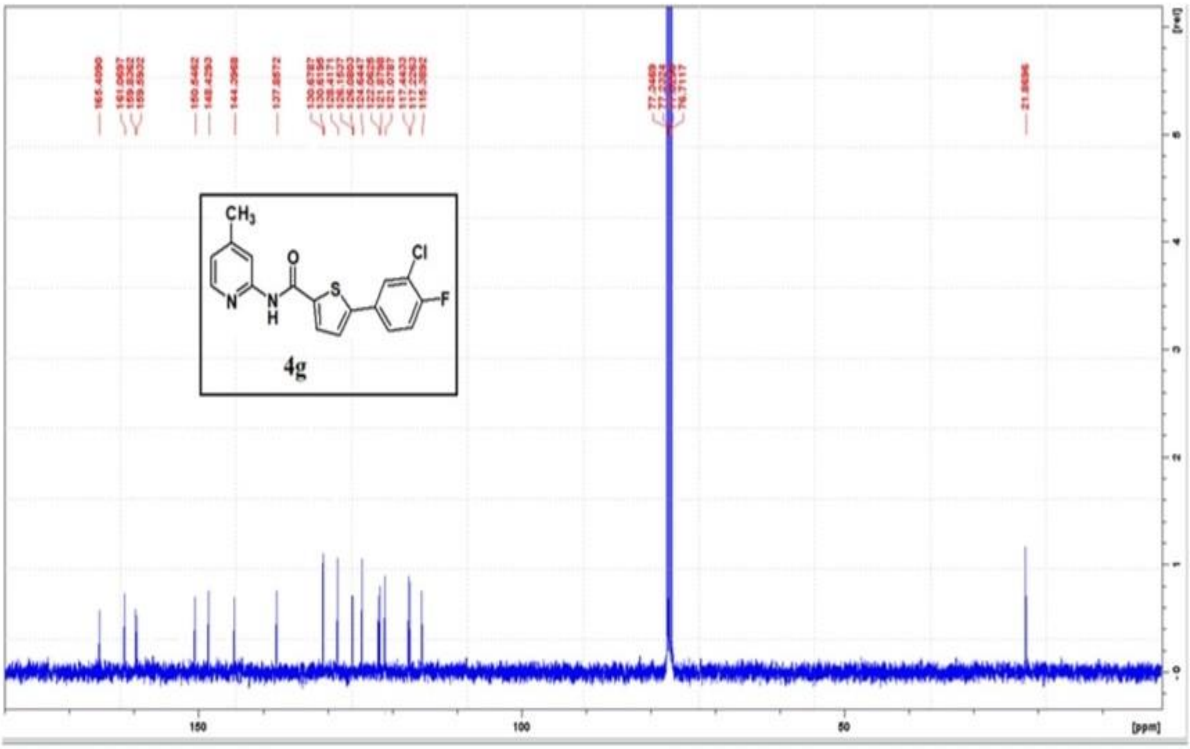

Figure S13: ^{13}C NMR (100 MHz, $CDCl_3$) of compound **4g**.

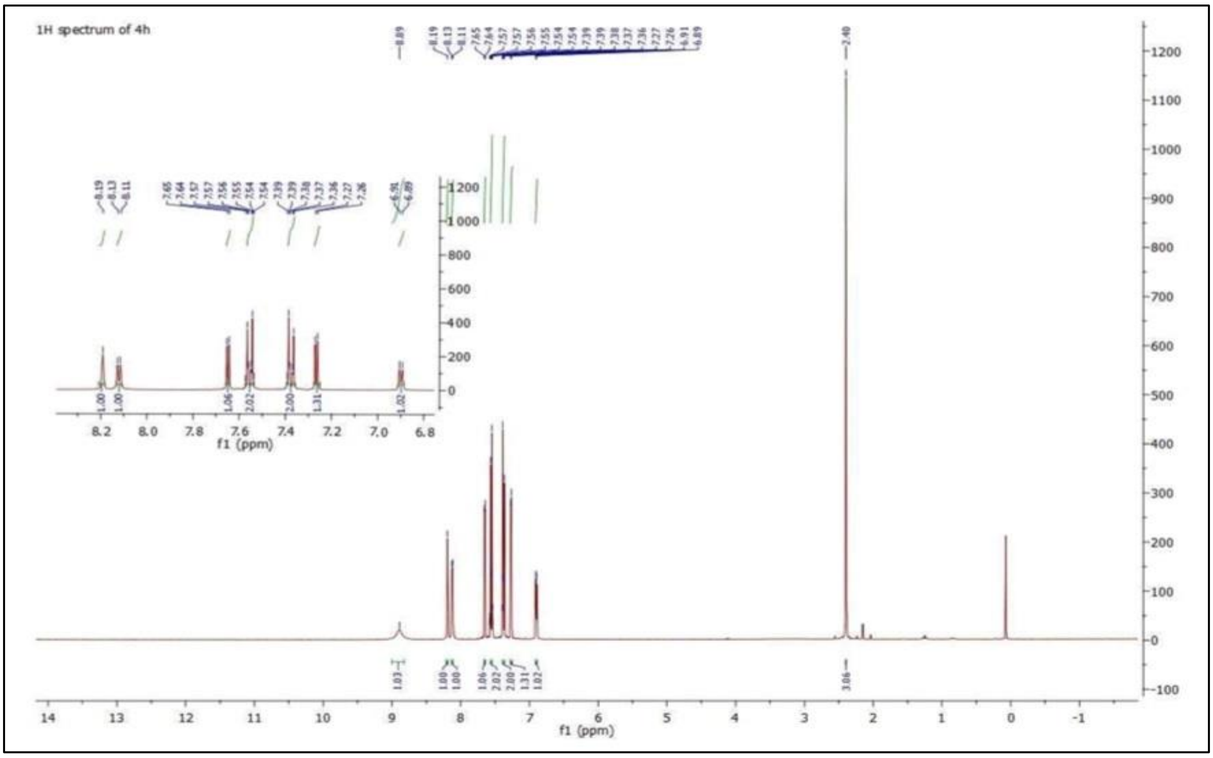

Figure S14: ^{1}H NMR (400 MHz, $CDCl_3$) of compound **4h**.
